# Severity of heterosubtypic influenza virus infection in ferrets is reduced by live attenuated influenza vaccine

**DOI:** 10.1038/s41541-021-00306-7

**Published:** 2021-03-29

**Authors:** Anthony C. Marriott, Karen E. Gooch, Phillip J. Brown, Kathryn A. Ryan, Nicola J. Jones, Natasha Merredew, Nathan Wiblin, Oliver Dibben, Helen Bright, Bassam Hallis, Catherine J. Whittaker, Miles W. Carroll

**Affiliations:** 1 National Infection Service, Public Health England, Porton Down, Wiltshire, UK; 2 Flu-MSAT, Biopharmaceutical Development, R&D, AstraZeneca, Liverpool, UK

**Keywords:** Immunology, Microbiology

## Abstract

Live attenuated influenza vaccine (LAIV) is widely used to protect humans from seasonal influenza infection, particularly in children. In contrast to inactivated vaccines, the LAIV can induce both mucosal and cellular immune responses. Here we show that a single dose of monovalent H1N1pdm09-specific LAIV in the ferret model is fully protective against a subsequent wild-type H1N1pdm09 challenge, and furthermore reduces the severity of disease following challenge with a different influenza A subtype (H3N2). The reduced severity comprised reductions in weight loss and fever, as well as more rapid clearance of virus, compared to non-vaccinated H3N2-challenged ferrets. No H3N2-neutralizing antibodies were detected in vaccinated ferret sera. Rather, heterosubtypic protection correlated with interferon-gamma+ (IFN-γ+) T-cell responses measured in peripheral blood and in lung lymphocytes. The IFN-γ+ cells were cross-reactive to H3N2 virus even when obtained from vaccinated animals that had never been exposed to H3N2 virus. We believe this study provides compelling evidence that the LAIV can provide a significant reduction in infection and symptoms when challenged with heterosubtypic influenza strains not included in the LAIV, highlighting the importance of cross-reactive T-cells in the design of a universal influenza vaccine.

## Introduction

The generation of “universal” influenza vaccines is a global research priority due to the challenges posed by the rapid evolution of influenza A and B viruses. Antigenic drift leads to the requirement to update virus strains included in vaccines on an annual basis for both Northern and Southern Hemispheres, with the A/H3N2 component being updated the most frequently^1^. Antigenic shift in influenza A results in viruses that have surface antigens to which the human population has no prior immune experience, and which are not contained in seasonal vaccines, leading to the possibility of a pandemic. Antigenic shift can arise when influenza A viruses from different species reassort to generate novel subtypes with the capacity to infect humans (as in the 1957 and 1968 pandemics), or when novel subtypes cross a zoonotic species barrier to infect humans (as in the 1918 pandemic).

Various strategies are being employed to create a vaccine that will induce broad(er) protection, thus reducing the need for annual updating, and with potential use against a novel pandemic influenza virus. It is generally agreed that both B-cell and T-cell immune responses will be required^2–4^. Of the seasonal vaccines currently widely used, the only replication-competent vaccines are the live attenuated influenza vaccines (LAIVs), which are based upon attenuated, temperature-sensitive master donor strains^5,6^. The surface proteins (HA and NA) are updated annually in the same way as traditional inactivated and subunit influenza vaccines, in order to match the antigens predicted to be circulating in the following influenza season. Currently the LAIV, which is used extensively to vaccinate children in the UK and other European countries, is a quadrivalent formulation comprising an H1N1, an H3N2, and two influenza B strains. In contrast to inactivated vaccines, LAIV replicates in the upper respiratory tract (URT) and generates a protective T-cell response in addition to the antibody response. In the ferret model, a trivalent LAIV containing the 2009 H1N1 attenuated virus reduced replication of a subsequent heterologous 2006 H1N1 challenge virus^7^, attributed in part to the presence of interferon-gamma-positive (IFN-γ+) T-cells, which were detected in PBMCs 7 days post-vaccination. Similarly, LAIV containing the 2007 H1N1 attenuated virus partially protected ferrets against disease induced by a 2009 H1N1 virus challenge, despite the lack of detectable neutralizing antibodies directed against the 2009 virus^8^.

Studies in humans have shown induction of CD4+ and CD8+ T-cells by the LAIV, with vaccine efficacy in children correlated to IFN-γ+ T-cell responses in peripheral blood mononuclear cells (PBMCs)^9–11^. T-cell responses were shown to exhibit broader reactivity against H3N2 drift variants than the antibody response did^12^. However, it has not been demonstrated that this T-cell response would protect against subtypes of influenza A virus not present in the vaccine. It has been shown that pre-existing T-cell memory in the population provided a significant level of cross protection during the 2009 influenza pandemic^13,14^, with the best correlate of protection being IFN-γ + CD8+ T-cells^14^. In a human volunteer challenge study using H3N2 or H1N1 viruses, the level of pre-existing CD4+ T-cells correlated with reduced disease^15^.

We have previously demonstrated that a mild infection with an H1N1pdm09 virus in the ferret model induces protective immunity against a heterosubtypic H3N2 challenge 4 weeks later^16^. This partial protection was not mediated by antibodies, but was correlated with a broadly reactive IFN-γ+ T-cell response. In this study, we demonstrate for the first time that vaccination with a monovalent H1N1 LAIV can provide a similar level of cross-protection against an H3N2 virus challenge.

## Results

### Study design

**Fig. 1:**
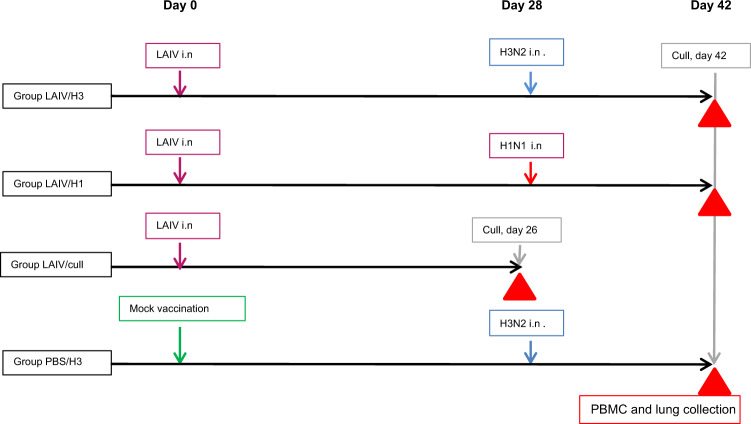
Outline of study design. Blood samples for serum and antigen stimulation, and nasal washes, were collected as described in “Methods”.

Ferrets were divided into 4 groups of 6, as shown in Fig. 1. Three groups of ferrets were vaccinated with LAIV on day 0 or received PBS mock-vaccination (group PBS/H3). Four weeks later ferrets were either culled (group LAIV/cull) or challenged with 100 plaque-forming units (PFU) of wild-type H1N1pdm09 virus (group LAIV/H1) or H3N2 virus (groups LAIV/H3 and PBS/H3). The remaining animals were culled 14 days post-infection (dpi). Virus shedding was monitored daily following LAIV vaccination and wild-type challenges.

### Monovalent LAIV vaccination induces strong H1N1-specific humoral immune response after a single dose

**Fig. 2:**
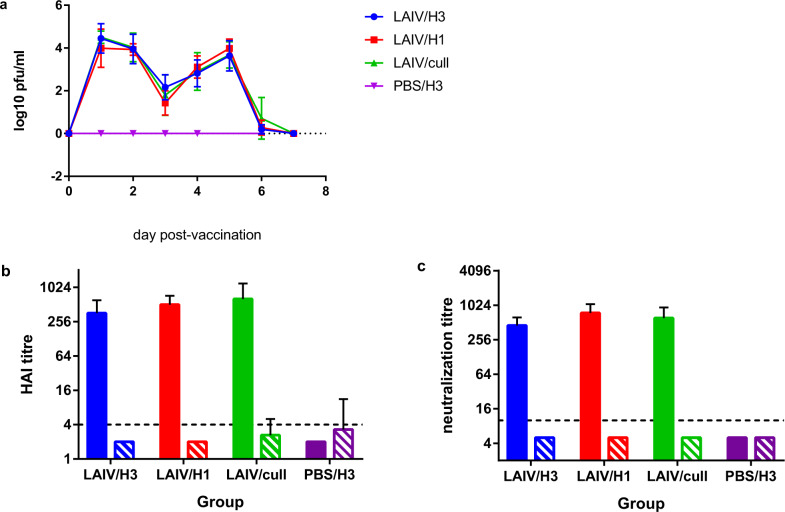
Vaccination with LAIV induces virus shedding and sero-conversion. **a** Virus titres in nasal wash following intra-nasal vaccination with LAIV. Day -3 samples are plotted as day 0. Points show group mean and standard deviation (*n* = 6). **b** Serum HAI titres to H1N1 and H3N2 viruses 24 days post-vaccination. **c** Serum microneutralization titres to H1N1 and H3N2 viruses 24 days post-vaccination. For (**b**) and (**c**), Solid bars: tested with H1N1 virus; hatched bars: tested with H3N2 virus. Dashed lines show limits of detection.

All vaccinated ferrets shed LAIV in their nasal wash for 5-6 days post-vaccination (Fig. 2a). No weight loss or fever was observed following vaccination (Supplementary Figs. 1 and 2), confirming the LAIV was attenuated in the ferret model. An inflammatory cell response was noted in nasal washes of vaccinated ferrets (Supplementary Fig. 3), similar to that reported previously for intra-nasal influenza A infections^16–18^. All ferrets sero-converted by day 24 post-vaccination (mean H1-specific HAI titres of ≥160), but no H3-specific antibody responses could be detected (Fig. 2b), except one ferret in the mock-vaccinated group showed an H3-specific titre of 40. All ferrets had titres of ≤8 prior to vaccination. Similarly, all ferrets showed neutralizing antibodies against H1N1 virus of ≥320 at day 24 post-vaccination, compared to the mock-vaccinated group which had titres ≤10 (Fig. 2c). No neutralizing antibodies to H3N2 virus could be detected. These data demonstrate that the LAIV replicated in the nasal cavity of the vaccinated ferrets without causing disease, and induced strong H1N1-specific antibody response.

### Monovalent LAIV induces an influenza-specific IFN-γ+ response in vaccinated ferrets

**Fig. 3:**
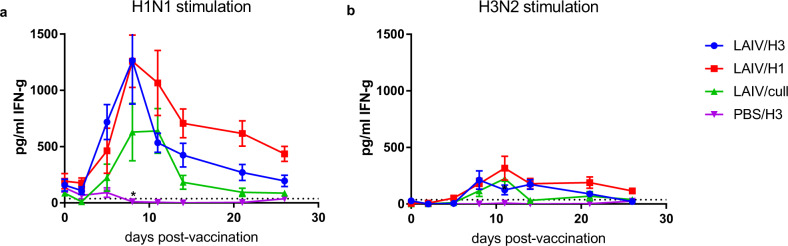
Cellular immune responses to vaccination with LAIV. IFN-γ response in antigen-stimulated whole blood was determined by ELISA. Blood was stimulated with wild-type (**a**) H1N1 or (**b**) H3N2 viruses. Points show mean and standard error. The black line (All LAIV) shows the mean of all LAIV-vaccinated ferrets. The dotted horizontal line shows the limit of quantitation. *PBS/H3 group was significantly lower than the vaccinated groups on (**a**) day 8 and (**b**) day 11 (Mann–Whitney test, *P* < 0.0001, *n* = 6).

Serial heparinized blood samples were taken before and after vaccination for stimulation with influenza H1N1 and H3N2 antigens, followed by quantitation of released IFN-γ (Fig. 3). Stimulation with H1 antigen showed peak response to LAIV on day 8 post-vaccination (Fig. 3a), with very little response in the mock group PBS/H3. Stimulation with H3 antigen shows peak response to LAIV on day 11, with very little response in the mock group PBS/H3 (Fig. 3b). These data demonstrate that the monovalent LAIV induced a cross-reactive IFN-γ+ response in the vaccinated ferrets.

### Monovalent LAIV vaccination induces protection against heterologous (H3N2) as well as homologous (H1N1) influenza virus infection

**Fig. 4:**
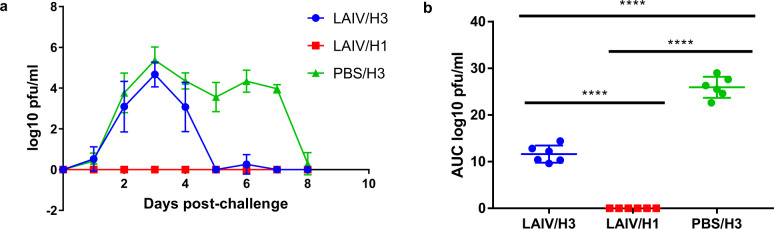
Nasal wash virus titres following challenge with wild-type viruses. **a** Mean and SD following challenge. **b** Area under the curve (AUC) analysis for each group. LAIV/H3 group shed significantly less virus than control group PBS/H3 (one-way ANOVA, *P* < 0.0001, *n* = 6).

Vaccination with H1N1 LAIV completely protected against homologous challenge (group LAIV/H1), i.e., there was no detectable virus replication (Fig. 4) and no observed signs of disease. Vaccination with H1N1 LAIV significantly reduced the duration of shedding and amount of virus shedding following heterologous H3N2 infection (group LAIV/H3) (Fig. 4a, b). Virus shedding fell to undetectable levels in the majority of ferrets in group PBS/H3 on 8 dpi whereas group LAIV/H3 fell to undetectable levels on 5 dpi. The mean duration of shedding (i.e. number of days with detectable infectivity in the nasal wash fluid) in the two groups was 7.0 ± 0.6 days for group PBS/H3 and 3.8 ± 0.8 days for LAIV/H3, which is a significant reduction (two-tailed Mann–Whitney test, *P* = 0.002). The peak of H3N2 virus shedding was at 3 dpi in both PBS/H3 and LAIV/H3 groups, and was of a very similar peak titre.

**Fig. 5:**
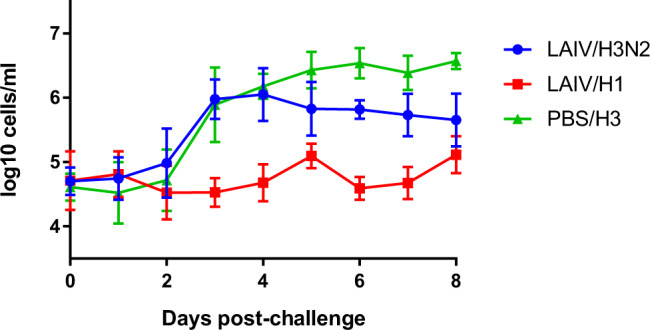
Nasal wash cell counts following challenge with wild-type viruses. Points show group mean and standard deviation. The increase in cell count represents the inflammatory response in the nasal cavity following infection^20^. Samples taken on day 26 post-vaccination are plotted as 0 dpi. The LAIV/H3 group is significantly different from the PBS/H3 group (*P* = 0.04), and both groups were significantly different from the LAIV/H1 group (*P* < 0.0001; AUC, one-way ANOVA with Tukey’s multiple comparisons test, *n* = 6).

Inflammatory response^17–20^ in vaccinated, H3N2-challenged ferrets (LAIV/H3) was significantly reduced relative to mock-vaccinated, H3N2-challenged ferrets (PBS/H3) (Fig. 5). The response in vaccinated ferrets matched the mock-vaccinated group until 4 dpi, then dropped off more rapidly. The homologous challenge group (LAIV/H1) showed no rise above baseline.

**Fig. 6:**
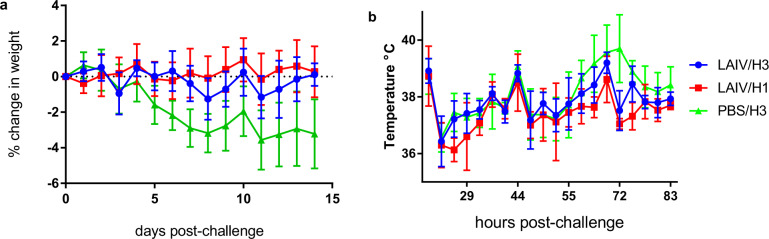
Protection from clinical disease conferred by LAIV vaccination. **a** Loss of bodyweight, normalised to day of challenge. **b** Temperature post-challenge. Group means and SD are plotted (*n* = 6).

Mock-vaccinated ferrets lost weight following H3N2 challenge, but LAIV-vaccinated ferrets lost little or no weight following either H1N1 or H3N2 challenge (Fig. 6a). Comparison of weights by the area under the curve (AUC) showed both homologous (*P* = 0.004) and heterologous (*P* = 0.02) groups had significantly reduced weight loss compared to unvaccinated group PBS/H3 (one-way ANOVA with Tukey’s correction, *n* = 6; Supplementary Fig. 4a).

Group PBS/H3 showed a characteristic rise in temperature from around 55 h post-infection (Fig. 6b), seen consistently with low-dose H3N2 infection in the ferret model^16^. Both groups LAIV/H1 and LAIV/H3 showed significantly reduced temperature relative to the PBS/H3 group (AUC from 55 to 83 h, one-way ANOVA, *P* = 0.0008 and 0.04 respectively, *n* = 6; Supplementary Fig. 4b).

Clinical signs following challenge comprised sneezing and inactivity; no clinical signs were observed in the homologous challenge group LAIV/H1. The heterosubtypic challenge group LAIV/H3 showed very similar signs to the control group PBS/H3 (Mann–Whitney test, *P* = 0.86, *n* = 6).

Taken together these data demonstrate partial protection in the LAIV/H3 group, despite the absence of H3-specific antibody response (Fig. 2).

### Immune responses following homologous and heterologous challenges in vaccinated ferrets

**Fig. 7:**
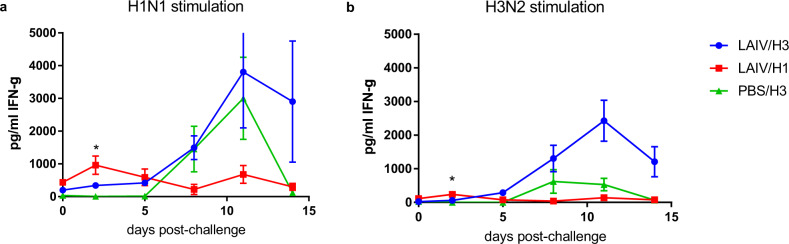
Cellular immune responses to virus challenge in vaccinated and unvaccinated animals. IFN-γ response in antigen-stimulated whole blood was determined by ELISA. Blood was stimulated with wild-type **a** H1N1 or **b** H3N2 viruses. Points show mean and standard error. Samples taken 2 days before the challenge (equivalent to day 26 post-vaccination) are plotted as day 0. *Group LAIV/H1 day 2 values were significantly higher than the other two groups for both H1 stimulation (**a**; 1-way ANOVA with Tukey’s multiple comparisons test, *P* = 0.003, *n* = 6) and H3 stimulation (**b**; *P* = 0.006).

Serial heparinized blood samples were taken for stimulation with influenza H1N1 and H3N2 antigens, followed by quantitation of released IFN-γ (Fig. 7). In addition, lung lymphocytes were prepared from ferrets for IFN-γ ELISpot at the termination of each group, as outlined in Fig. 1. H3N2 challenge gave a strong response in both vaccinated (group LAIV/H3) and control (group PBS/H3) ferrets, peaking 11 dpi. Homologous challenge group LAIV/H1 shows a more transient response peaking 2 dpi, and day 2 values were higher than the other two groups for both H1 and H3 stimulation. There was a trend to a higher response 2 dpi in group LAIV/H3 vs PBS/H3, but it was not significant (one-way ANOVA, *P* = 0.35–0.64, *n* = 6).

**Fig. 8:**
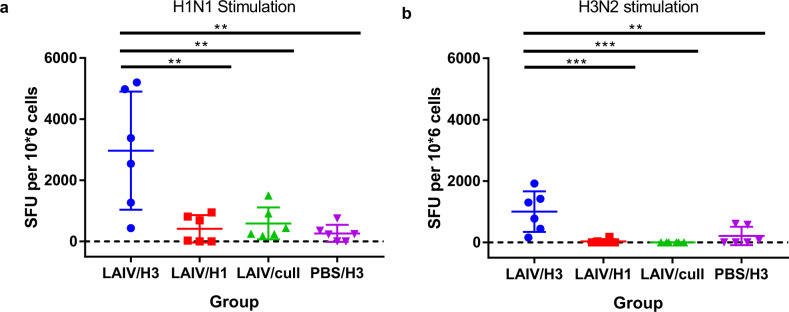
Cellular immune response in lungs, measured by IFN-γ ELISpot. Purified lung lymphocytes were stimulated with **a** H1N1 or **b** H3N2 viruses. Lungs were collected at 14 dpi (groups LAIV/H3, LAIV/H2 and PBS/H3) or at 26 days post-vaccination (group LAIV/cull). Lines show group mean and SD. Groups were compared by one-way ANOVA with Tukey’s multiple comparisons test, *n* = 6. **P < 0.01; ***P < 0.001.

Lung lymphocytes were stimulated with H1N1 and H3N2 viruses, and IFN-γ secreting cells were visualised by ELISpot (Fig. 8). Group LAIV/cull represents the lung status 2 days prior to infection in vaccinated ferrets. Comparing group LAIV/cull to group LAIV/H1 shows little difference (*P* > 0.9), whereas comparing to group LAIV/H3 shows a significant increase in both H1-specific (*P* = 0.004) and especially H3-specific T-cells (*P* = 0.0006, *n* = 6) by 14 dpi. Low signals in group PBS/H3 correlate with the low peripheral blood ELISA values at 14 dpi. For both H1N1 and H3N2 stimulation, the heterologous challenge group LAIV/H3 showed higher spot-forming unit (SFU) counts than any of the other groups.

These data show evidence for a rapid memory IFN-γ+ response to homologous challenge in group LAIV/H1, as well as an increase in both H1-stimulated and H3-stimulated responses in the LAIV/H3 group.

## Discussion

LAIVs have previously been shown to induce greater T-cell responses than inactivated influenza vaccines, both in humans and in the ferret model^7,10,11,21^. In humans these T-cells have shown cross-reactivity to H1N1 and H3N2 virus strains not contained in the vaccine^22^. This may lead to a protective immune response with broader specificity than that comprising the antibody response only, as the T-cells may be directed against the more highly conserved internal proteins of the virus (e.g. nucleoprotein NP). Several studies have shown that the LAIV can induce protective antibodies with a broader specificity than the corresponding inactivated vaccine, but these responses are limited to cross-protection between strains within a subtype^6,9,23^. IFN-γ+ T-cell responses to wild-type influenza infections give at least limited protection to subsequent heterosubtypic infections^16,24,25^. Pre-existing T-cell responses to influenza gave protection against severe disease during the 2009 H1N1 pandemic, which despite not being a novel subtype, was highly divergent from the H1N1 viruses widely circulating in the population at that time^13,14^. These T-cell responses were characterised as being predominantly NP-specific^13^ or predominantly directed to NP, M1 and PB1 proteins^14^.

We have demonstrated in this study that a single human dose of monovalent H1N1 LAIV is highly attenuated in ferrets (no fever, weight loss or respiratory disease), and induces a strong neutralizing antibody response specific for the H1N1 subtype. Subsequent challenge with H1N1 resulted in sterilising immunity, i.e., no detectable virus replication and no clinical signs of disease. Although we did not have an unvaccinated, H1N1-infected control group in this study, we have extensive data to show the expected level of virus shedding, fever, weight loss and other clinical signs following low-dose H1N1 challenge^16–18^.

Importantly, we have shown that this monovalent H1N1 vaccination protects ferrets from a heterosubtypic H3N2 virus challenge, albeit to a lesser degree than seen with the homologous H1N1 virus challenge. This result confirms that the LAIV can induce a similar protective response to infection with the wild-type virus, despite the LAIV being restricted to the upper respiratory tract, in contrast to the wild-type H1N1 virus which also infects the lungs. Previous studies have shown that trivalent LAIV is able to protect against drift variants not contained in the vaccine^26,27^, however we made use of a monovalent LAIV to demonstrate heterosubtypic protection for the first time. The protection was characterised by a reduction in fever and weight loss, reduced nasal inflammatory response, and more rapid clearance of virus. The reduction in disease severity suggests that LAIVs may provide a level of protection against infection with heterosubtypic influenza strains in humans. The significant reduction in virus shedding after heterosubtypic infection may be important for onward transmission of the infection, although that was not investigated in this study.

No cross-neutralizing antibodies were detected in the vaccinated ferrets, and this is unsurprising as the immunodominant region of the virus is the HA “head” region, which is highly divergent between H1 and H3 HA’s. Instead, we observed influenza-specific IFN-γ+ responses in peripheral blood which were inducible by H3N2 virus stimulation as well as by H1N1 stimulation, peaking 8-11 days post-vaccination but still detectable 26 days post-vaccination (Fig. 3). At this stage in the study (day 26) the ferrets had not been exposed to H3N2 virus, so it seems highly likely that cross-reactive IFN-γ+ T-cells were induced by the vaccination, given that H1N1 and H3N2 viruses share many conserved epitopes, particularly in the major internal proteins such as NP and M1^28^. Upon H3N2 challenge, a strong enhancement of the IFN-γ+ response was observed in the vaccinated ferrets (Fig. 7), indicative of a memory T-cell response. In the homologous challenge group, a significant IFN-γ+ response was observed 2 dpi, which may also indicate a rapid memory recall response, as observed previously in ferrets primed by wild-type H1N1 virus infection^16^. A similar response was observed using ELISpot to stimulate lung lymphocytes with H1N1 and H3N2 antigens, in that the LAIV/H3 group produced a stronger response than the PBS/H3 control group (Fig. 8), which could represent the difference between recall and primary responses, respectively. Taken together, these data suggest that the heterosubtypic cross-protection we observed is highly correlated with the cross-reactive IFN-γ+ T-cell response.

A comparatively weak IFN-γ response was observed in the lungs of the LAIV/H1 group. This is most likely due to the sterilising nature of the immunity to H1N1 virus induced by the LAIV, which prevented detectable virus replication in the nasal cavity, thus minimising the amount of virus available to reach the lungs and trigger a cellular immune response. A similar effect was observed in a previous study, in which the immunity induced by a wild-type infection minimised the lung response to a subsequent infection with the same virus^16^.

The interval between vaccination and virus challenge used in this study was 4 weeks. It would be of great interest to know the duration of the heterosubtypic protection, which could potentially ameliorate disease for much longer periods if it is driven by memory T-cells. There is evidence from the ferret model that heterosubtypic protection may last up to 18 months, although that study used wild-type virus rather than LAIV to induce protection^29^.

Vaccines specifically designed to elicit T-cell responses to influenza virus are in clinical trials as putative “universal” influenza vaccines^30–32^, targeting NP, M1 and M2 proteins. In studies of trivalent LAIV in children, PBMCs were found to respond to NP and M1 peptides^11,22^. NP-specific T-cells were also reported in a ferret study with trivalent LAIV^33^. Future studies will be designed to determine the peptide specificity of the cross-reactive T-cells responsible for protection seen in this study, and this data could inform the design of universal vaccines intended to protect in the case of the emergence of new subtype(s) of influenza A virus into the human population. Although T-cell epitopes are not likely to be identical between ferrets and humans, the data available to date suggest that overall T-cell responses to influenza infection in both humans and ferrets are predominantly directed to internal conserved proteins such as NP and M1. Ultimately a vaccine that only induces T-cell responses is unlikely to be fully protective, since the target of T-cells is virus-infected cells, which only become present once infection is already underway. Hence a combined approach using broadly reactive antibodies for protection and T-cells for more rapid clearance of virus seems the most likely to form the basis of a successful universal influenza vaccine.

## Methods

### Viruses and cells

Influenza A/California/04/09 (H1N1) and A/Perth/16/09 (H3N2) were obtained from the Centers for Disease Control and Prevention (CDC, Atlanta, USA), and National Institute for Biological Standards and Control (NIBSC, Potters Bar, UK), respectively). Monovalent H1N1 LAIV, containing the HA and NA genes from A/California/07/09, was supplied by AstraZeneca, Liverpool, UK. LAIV A/CA09 was propagated in the allantoic cavity of 10–11-day-old embryonated hens’ eggs (Charles River Laboratories, Wilmington, MA, USA). The six internal gene segments (PB2, PB1, PA, NP, M, and NS) were provided by cold-adapted, temperature-sensitive A/Ann Arbor/6/1960^34^. The HA and NA genes were derived from wild-type A/California/07/2009^35^.

Viruses were titrated in Madin-Darby Canine Kidney (MDCK) cells, obtained from European Collection of Cell Cultures (ECACC, Porton Down, UK). Virus titres were determined by plaque assay on MDCK cells under an agar overlay containing 1.8 µg/ml TPCK-trypsin (Merck Life Science, UK), followed by staining with crystal violet (wild-type viruses), or immuno-staining with an NP-specific monoclonal antibody^36^ for the LAIV. LAIV plaque assays were incubated at 33 °C for 3 days prior to staining with NP-specific antibody, followed by anti-mouse alkaline phosphatase and BCIP-NBT Plus reagent (Mabtech, Nacka, Sweden).

### Animals

Twenty-four female ferrets (*Mustela putorius furo*) were obtained from Highgate Farm, UK, and confirmed as seronegative for influenza H1N1pdm09, H3N2 and influenza B antibodies by haemagglutination-inhibition (HAI) assay before experiments commenced. Mean weight at vaccination was ~960 g. An identifier chip (Bio-Thermo Identichip, Animalcare Ltd, UK) was inserted subcutaneously into the dorsal cervical region of each animal. Animals were monitored for signs of disease (sneezing, nasal discharge, lethargy) twice daily (~8 h apart), and weight was recorded daily. The temperature was monitored twice daily using the chip, increased to seven times daily during the 3 days following challenge virus inoculations, to ensure any peak of fever was recorded. Animals were sedated by intramuscular injection of ketamine/xylazine (17.9 and 3.6 mg/kg bodyweight), prior to intranasal instillation of challenge virus (0.2 ml total, 0.1 ml per nostril) diluted in phosphate-buffered saline (PBS). Vaccination comprised 0.2 ml LAIV containing 10^7^ PFU virus. Nasal washes were obtained using 2 ml PBS. The experimental animal work described here was scrutinized and approved by the Animal Welfare and Ethical Review Body of Public Health England (Porton), as required by the UK Home Office Animals (Scientific Procedures) Act, 1986. The premises in which the work was conducted are approved under Home Office Certificate of Designation PCD70/1707. All methods involving ferrets were performed in accordance with the relevant guidelines and regulations.

### Serum antibody

Serum samples were titrated by HAI assay using 4 HA units per well of the relevant virus, followed by the addition of 0.5 % v/v chicken red blood cells. Selected sera were also titrated by microneutralization assay on MDCK cells^14^. In brief, virus and sera were incubated together for 2 h at 37 °C, then mixed with MDCK cells in a microtitre plate. 18–20 h later cells were fixed, and stained with a mouse anti-NP antibody, followed by goat anti-mouse horseradish peroxidase conjugate and o-phenylenediamine substrate.

### Isolation of PBMCs and lung mononuclear cells

Buffy coats containing lymphocytes were prepared from fresh heparin anti-coagulated blood by density separation on Histopaque 1083^18^. Cells were collected by centrifugation and re-suspended in an appropriate volume of cryomedia (90% foetal bovine serum, 10% DMSO) allowing the cells to be stored in liquid nitrogen in 1 ml aliquots at a concentration of 3 × 10^6^ to 1.3 × 10^7^ cells/ml. Dissected lungs were dissociated using a gentleMACS Tissue Dissociator (Miltenyi Biotec, UK)^18^. The tissue suspension was passed through two cell sieves (100 µm then 70 µm) and mononuclear cells were purified and frozen as described for PBMCs above. Red blood cells were removed from PBMC and lung MNC preparations using 5 min incubation in ACK Lysing Buffer (Gibco, ThermoFisher Scientific, UK).

### Viable cell counts

Viable cells in nasal washes, PBMCs and lung MNCs were counted using a NucleoCounter®NC-200 (ChemoMetec, Allerod, Denmark). Cell suspensions were loaded into Via-1 cassettes which use Acridine Orange and DAPI dyes to perform live/dead staining, according to the manufacturer’s instructions.

### Interferon-gamma (IFN-γ) ELISpot assay

Lung MNC were assessed for responses to A/California/07/2009 (H1N1) and A/Perth/16/2009 (H3N2)^18^. Both viruses were used at a multiplicity of infections of 0.08. Following overnight stimulation, IFN-γ expressing cells were detected using the Ferret interferon-gamma ELISpot kit with pre-coated plates (Mabtech, Nacka, Sweden). Results from duplicate tests were averaged. Data were analysed by subtracting the mean number of spots in the control wells (cells and allantoic fluid) from the mean counts of spots in wells with cells and antigen.

### Ferret IFN-γ enzyme-linked immunosorbent assay (ELISA)

Heparinised whole blood was diluted 1:10 with serum-free RPMI 1640 medium and incubated with either A/California/07/2009 (H1N1) or A/Perth/16/2009 (H3N2) allantoic fluids (1.6 × 10^6^ PFU). Phytohemagglutinin PHA-M (Sigma-Aldrich, Dorset, UK) was used as a positive control and egg allantoic fluid was used as a negative control. Blood was stimulated for 4 days at 37 °C, after which plasma supernatants were collected and cryopreserved at −80 °C. The Ferret IFN-γ ELISA Development Kit (ALP) (Mabtech, Nacka, Sweden) was used to determine the quantity of IFN-ɣ secreted by cells in the blood as described^18^.

### Statistical analysis

Statistical analyses were performed with GraphPad Prism 7 (GraphPad Software, La Jolla, CA). All measurements were taken from distinct samples. *P*-values of <0.05 were considered significant.

### Reporting summary

Further information on research design is available in the Nature Research Reporting Summary linked to this article.

## Supplementary information

Supplementary Information

Reporting Summary

## Data Availability

All data are included in the paper and its supplementary materials. Materials and access to raw data will be supplied by the authors upon reasonable request: enquiries to Anthony.marriott@phe.gov.uk.
