# Supplementary material for: Severity of heterosubtypic influenza virus infection in ferrets is reduced by live attenuated influenza vaccine
Source: NPJ Vaccines. 2021 Mar 29;6:43. doi: 10.1038/s41541-021-00306-7 (PMC8007727; doi:10.1038/s41541-021-00306-7)
Supplement: Supplementary file 1 — Supplementary Information [file 41541_2021_306_MOESM1_ESM.pdf]

Supplementary Figures.

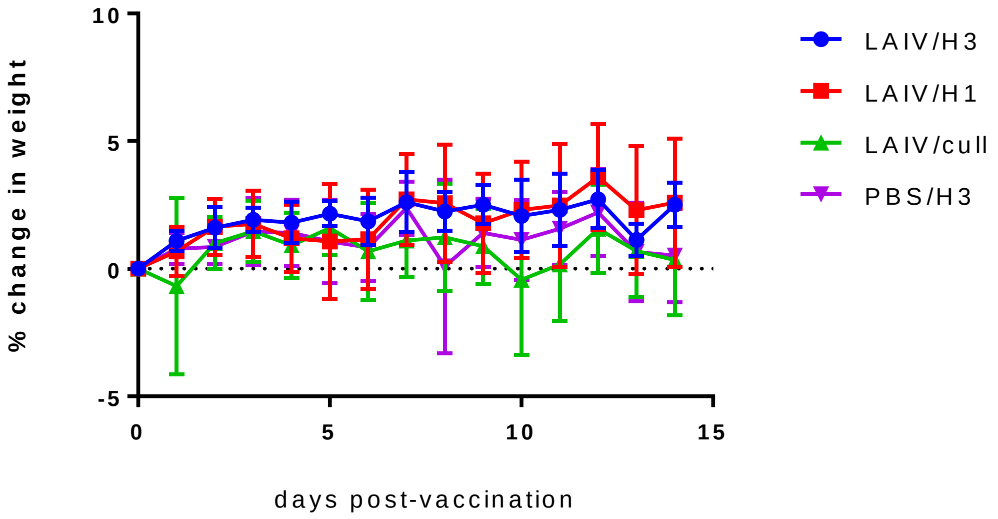

Supplementary Fig. 1. Weight change after LAIV vaccination. Group mean and SD are shown (n = 6).

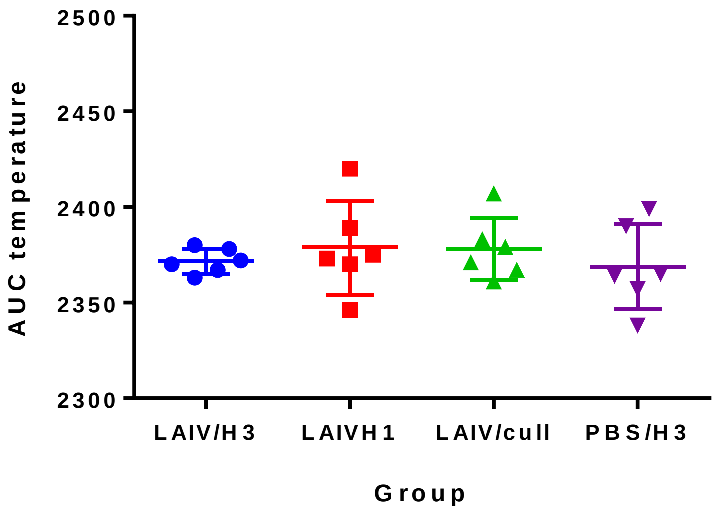

Supplementary Fig. 2. Area under the curve (AUC) analysis for temperature after LAIV vaccination (n = 6).

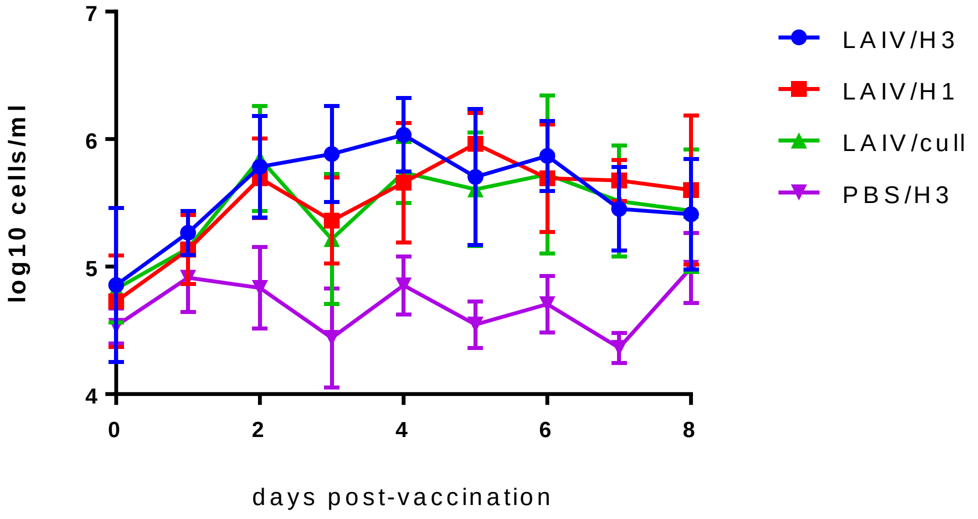

Supplementary Fig. 3. Nasal wash cell counts following intra-nasal vaccination with LAIV. Vaccination with LAIV induces an inflammatory response in the nasal cavity which remains above baseline for ≥ 8 days. Group mean and SD are shown (n = 6).

A.

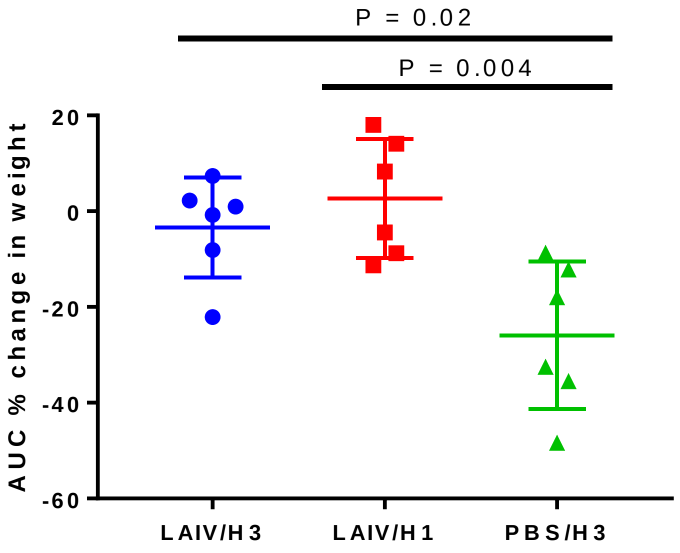

B.

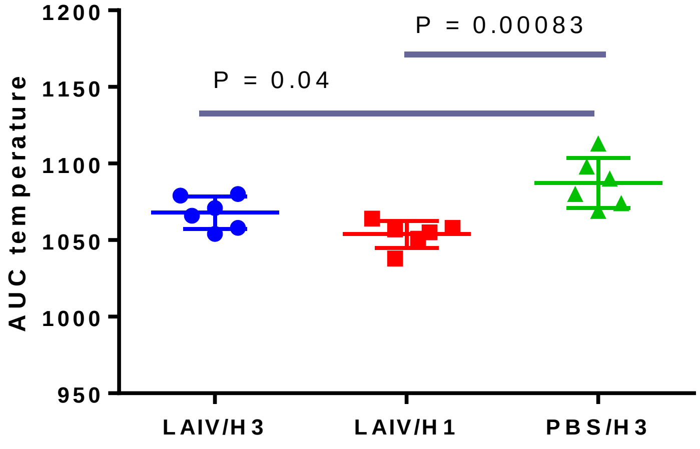

Supplementary Fig. 4. Area under the curve (AUC) analysis for (**A**) weight change and (**B**) temperature after wild-type H1N1 or H3N2 challenge. Groups were compared using 1-way ANOVA with Tukey's correction for multiple groups (n = 6).
