# Supplementary material for: Severity of heterosubtypic influenza virus infection in ferrets is reduced by live attenuated influenza vaccine
Source: NPJ Vaccines. 2021 Mar 29;6:43. doi: 10.1038/s41541-021-00306-7 (PMC8007727; doi:10.1038/s41541-021-00306-7)
Supplement: Supplementary file 2 — Reporting Summary [file 41541_2021_306_MOESM2_ESM.pdf]

nature research

Corresponding author(s): Anthony Marriott

Last updated by author(s): Feb 12, 2021

Reporting Summary

Nature Research wishes to improve the reproducibility of the work that we publish. This form provides structure for consistency and transparency in reporting. For further information on Nature Research policies, see our Editorial Policies and the Editorial Policy Checklist.

Statistics

For all statistical analyses, confirm that the following items are present in the figure legend, table legend, main text, or Methods section.

n/a	Confirmed	
☐	☒	The exact sample size (*n*) for each experimental group/condition, given as a discrete number and unit of measurement
☐	☒	A statement on whether measurements were taken from distinct samples or whether the same sample was measured repeatedly
☐	☒	The statistical test(s) used AND whether they are one- or two-sided<br>*Only common tests should be described solely by name; describe more complex techniques in the Methods section.*
☒	☐	A description of all covariates tested
☐	☒	A description of any assumptions or corrections, such as tests of normality and adjustment for multiple comparisons
☐	☒	A full description of the statistical parameters including central tendency (e.g. means) or other basic estimates (e.g. regression coefficient) AND variation (e.g. standard deviation) or associated estimates of uncertainty (e.g. confidence intervals)
☐	☒	For null hypothesis testing, the test statistic (e.g. *F*, *t*, *r*) with confidence intervals, effect sizes, degrees of freedom and *P* value noted<br>*Give P values as exact values whenever suitable.*
☒	☐	For Bayesian analysis, information on the choice of priors and Markov chain Monte Carlo settings
☒	☐	For hierarchical and complex designs, identification of the appropriate level for tests and full reporting of outcomes
☒	☐	Estimates of effect sizes (e.g. Cohen's *d*, Pearson's *r*), indicating how they were calculated

Our web collection on statistics for biologists contains articles on many of the points above.

Software and code

Policy information about availability of computer code

Data collection	Cell counts: Nucleoview NC-200 (Chemometec); Elispot counts Immunospot 5.0 Analyzer (Cellular Technology Ltd)
Data analysis	Graphpad Prism v 7; Microsoft Excel 2016

For manuscripts utilizing custom algorithms or software that are central to the research but not yet described in published literature, software must be made available to editors and reviewers. We strongly encourage code deposition in a community repository (e.g. GitHub). See the Nature Research guidelines for submitting code & software for further information.

Data

Policy information about availability of data

All manuscripts must include a data availability statement. This statement should provide the following information, where applicable:

- Accession codes, unique identifiers, or web links for publicly available datasets
- A list of figures that have associated raw data
- A description of any restrictions on data availability

All data is included in the paper and its supplementary materials.

Field-specific reporting

Please select the one below that is the best fit for your research. If you are not sure, read the appropriate sections before making your selection.

☒ Life sciences ☐ Behavioural & social sciences ☐ Ecological, evolutionary & environmental sciences

For a reference copy of the document with all sections, see nature.com/documents/nr-reporting-summary-flat.pdf

Life sciences study design

All studies must disclose on these points even when the disclosure is negative.

Sample size	Group size n = 6 chosen for ferrets based on previous published studies. Ferret numbers are limited by ethical and financial constraints.
Data exclusions	No data excluded.
Replication	Reproducibility between ferrets in a treatment group is shown in the Results. Reproducibility between assays was demonstrated by use of the appropriate positive and negative controls.
Randomization	Ferrets were assigned to groups to give similar starting weight distribution. Full randomisation is not possible as ferrets are raised in social groups and mixing these up will lead to animals fighting.
Blinding	Blinding was not used in this study. Animal handlers are aware which groups receive which intervention.

Reporting for specific materials, systems and methods

We require information from authors about some types of materials, experimental systems and methods used in many studies. Here, indicate whether each material, system or method listed is relevant to your study. If you are not sure if a list item applies to your research, read the appropriate section before selecting a response.

Materials & experimental systems

n/a	Involved in the study	
☐	☒	Antibodies
☐	☒	Eukaryotic cell lines
☒	☐	Palaeontology and archaeology
☐	☒	Animals and other organisms
☒	☐	Human research participants
☒	☐	Clinical data
☒	☐	Dual use research of concern

Methods

n/a	Involved in the study	
☒	☐	ChIP-seq
☒	☐	Flow cytometry
☒	☐	MRI-based neuroimaging

Antibodies

Antibodies used	Ferret IFN-g monoclonal used in Elispot and ELISA assays: clone MTF19 as part of Mabtech kits (3112-1A-6). Influenza Anti-NP monoclonal used in LAIV plaque assays and microneutralization tests: clone AA5H (Invitrogen MA1-23054).
Validation	MTF19 is validated using purified ferret IFN-g controls in each assay. AA5H is used to detect presence of influenza virus antigen and is controlled using uninfected cells in each assay.

Eukaryotic cell lines

Policy information about cell lines

Cell line source(s)	MDCK cells from ECACC, 84121903
Authentication	All ECACC animal cell lines are authenticated by DNA barcoding
Mycoplasma contamination	Negative for Mycoplasma by PCR test.
Commonly misidentified lines (See ICLAC register)	None

Animals and other organisms

Policy information about studies involving animals; ARRIVE guidelines recommended for reporting animal research

Laboratory animals	Ferrets (Mustela putorius furo). Female, 4-6 months.
Wild animals	The study did not involve wild animals.
Field-collected samples	The study did not involve field-collected samples.
Ethics oversight	Ethical oversight by the Animal Welfare and Ethical Review Body of Public Health England (Porton), as required by the UK Home Office Animals (Scientific Procedures) Act, 1986.

Note that full information on the approval of the study protocol must also be provided in the manuscript.
